# Profile of patients presenting at a low vision clinic in a developing country

**DOI:** 10.1186/1471-2415-12-31

**Published:** 2012-07-30

**Authors:** Bolutife Olusanya, Godfrey Onoja, Waheed Ibraheem, Charles Bekibele

**Affiliations:** 1 Department of Ophthalmology, University College Hospital, Ibadan, Nigeria

**Keywords:** Low vision, Patient characteristics, Developing countries

## Abstract

**Background:**

Low vision is an important public health problem; however, very few low vision clinics are available to address the needs of low vision patients in most developing countries. The purpose of this study was to describe the characteristics of patients attending the low vision clinic of a Nigerian tertiary hospital.

**Methods:**

This was a prospective cross sectional study of all new patients seen at the low vision clinic over a 36 month period. Patients were administered with a structured questionnaire, and were examined and tested with low vision devices by the attending low vision specialist. Information on the demographic and clinical characteristics of the patients was recorded.

**Results:**

A total of 193 new patients seen during the period were studied. The mean age was 41.4 years, and their ages ranged between 6 and 90 years with a male to female ratio of 1.9:1. Majority (58%) were aged below 50 years, 23.3% were children (≤15 years), while 21.8% were elderly patients (≥65 years). The commonest cause of low vision was retinitis pigmentosa (16.6%); 14.5% had age related macular degeneration (ARMD); 9.8% had albinism; while only 1% had diabetic retinopathy. ARMD (45.2%) was the commonest cause in the elderly patients, while albinism (24.4%) and optic atrophy (24.4%) were the commonest in children.

**Conclusion:**

The demographic and clinical characteristics of low vision patients seen in this clinic are similar to that of patients in other developing countries, but different from those in developed countries. Elderly patients and females may be under-utilising low vision services. There is a need for further research into the determinants of low vision service utilisation in developing countries. This would further aid the planning and delivery of services to low vision patients in these countries.

## Background

A person with low vision is one who has impaired visual function despite treatment of eye disease and/ or correction of refractive error, and has reduced visual acuity in the better eye which is less than 6/18 but better than light perception (LP) or a visual field constriction to less than 10°, but who uses or is potentially able to use vision for the planning and/ or execution of a task [1]. This definition of low vision excludes individuals whose visual acuity could be improved by surgical and/or medical treatment and refers to functional vision. It differs from other definitions of low vision in the literature which include all individuals presenting with impaired vision regardless of the cause, potential for treatment and the ability to use residual vision. The term “functional low vision” has been used to represent this definition in a bid to the avoid confusion with other definitions [2-4]. People with functional low vision require assessment for low vision interventions [2], and such patients are the focus of this article.

Functionally, low vision is characterised by irreversible visual loss and a reduced ability to perform many daily activities, such as recognising people in the street, reading blackboards, writing at the same speed as peers, and playing with friends [5]. It is an important public health problem [6]; and provision of low vision services is one of the priorities in the global initiative, VISION 2020—The Right to Sight [2,7].

Based on figures from the Nigerian National Blindness and Visual impairment survey, it is estimated that approximately 800,000 individuals have functional low vision in Nigeria [3]. The challenge of providing low vision services for such a large population is enormous and requires proper planning and efficient use of available resources. It is, therefore, important to collect and analyse clinical data from patients with functional low vision in order to deliver appropriate low vision care.

Majority of available data on people with low vision are derived from population surveys which were not specifically designed to study functional low vision while clinical studies which give more detailed information on patients actually attending low vision clinics are few. To the best of our knowledge, there are no previous reports of such clinical studies from Nigeria, as there are very few low vision centres available in the country. This study was carried out to describe the demographic and clinical characteristics of patients presenting to the low vision clinic of a university teaching hospital in south-western Nigeria. We believe that the information about patients who actually attend low vision clinics would be useful for planning and delivery of effective low vision services.

## Methods

This was a prospective cross sectional study of all new patients seen at the low vision clinic of University College Hospital (UCH), Ibadan between August 2005 and July 2008.

### Study location

UCH is a university teaching hospital and is a major provider of tertiary healthcare to the south-western region of Nigeria. The low vision clinic was established with the aid of a World Health Organization assisted project in 2005, and services commenced in August of the same year. It is located in the eye clinic of the hospital with a dedicated room equipped for evaluation and testing of patients with low vision.

### Study population

Clients who attend the low vision clinic comprise of patients who have been treated at the main eye clinic for various ailments but whose vision needs were not adequately met by conventional methods in accordance with the Bangkok definition of low vision [1]. Thus, most subjects with operable cataracts are not routinely referred to the low vision clinic and were not included in this study. Other sources of referral include private and state-owned eye clinics in the southwest region of Nigeria. All patients presenting to the low vision clinic are seen by an ophthalmologist and/or an optometrist who has received subspecialty training in low vision services. Low vision devices are available for purchase by clients as soon as they are prescribed.

All the patients seen during the study period were administered with a structured questionnaire, and were examined and tested with different low vision devices by the attending low vision specialist. Information on the demographic and clinical characteristics of the patients was recorded. Visual acuity (VA) was assessed with the use of Early Treatment Diabetic Retinopathy Study (ETDRS) charts and recorded in logarithm of the minimum angle of resolution (logMAR) units for distance vision and metric units (M) for near vision. Distance visual acuity of counting fingers, hand motion, light perception (LP) and nil light perception (NLP) were assigned logMAR values of 1.9, 2.3, 2.7 and 3.0 respectively [8-10]. Colour vision was tested by correct identification of coloured pencils.

The study protocol adhered to the tenets of the Declaration of Helsinki for research involving human subjects and verbal informed consent was obtained from study participants. Ethical approval was obtained from the ethics committee of UCH. The data was analysed with the use of Statistical Package for Social Sciences version 17 software (SPSS Inc., Chicago, Illinois, United States of America.)

## Results

A total of 193 new patients presented and were seen at the low vision clinic during the study period. The mean age was 41.4 (±23.8) years. Their ages ranged between 6 and 90 years. Majority (65.8%) of the patients were males with a male to female ratio of 1.9: 1. The 10–19 years age group had the largest proportion with 19.2% of patients; followed by the 60–69 years age group (16.6%). The age distribution of the patients is shown in Table 1.

**Table 1:** Age distribution of low vision patients

**Age group (years)**	**Frequency (n)**	**Percent (%)**
0 – 9	14	7.3
10 – 19	37	19.2
20 – 29	24	12.4
30 – 39	13	6.7
40 – 49	24	12.4
50 – 59	21	10.9
60 – 69	32	16.6
70 – 79	22	11.4
80 and above	6	3.1
Total	193	100.0

Forty five (23.3%) patients were children (aged 15 years or less), 106 (54.9%) were aged between 16 and 64 years, while 42 (21.8%) of them were elderly patients (65 years and older). The proportion of females in each of these categories decreased from the youngest to the oldest [χ^2^ = 5.66; p = 0.059]. The mean age of the male patients was 43.6 years while that of females was 37.0 years [t =1.84; p = 0.067].

With regards to the main presenting complaint, 103 (53.4%) patients complained of both poor distance and near vision indicating that both distance and near vision were of equal importance to them. Thirty nine (20.2%) patients said their major problem was with distance vision, while 38 (19.7%) reported that their main complaint was poor near vision. Three patients (1.6%) had night blindness as the main symptom while one (0.5%) patient was mainly concerned about restriction of his visual field.

Amongst the patients with poor distance vision as their main problem, 41.0% were children and 7.7% were elderly patients; but among those with poor near vision as their major complaint, only 13.2% were children while 36.8% were elderly patients [χ^2^ = 12.89; p = 0.002].

The median presenting distance VA in the better eye was 1.20 logMAR with an interquartile range (IQR) of 0.90 – 1.36 logMAR. The median distance VA of the right eyes was 1.30 logMAR (IQR: 1.00 – 1.52); the median for the left eyes was also 1.30 logMAR (IQR: 0.97 – 1.50).

The right eye had the better VA in 60 (31.1%) patients, the left eye was better in 54 (28%) patients, while the visual acuity was equal in both eyes of 79 (40.9%) patients.

The median near visual acuity was 2.5 Metric units (IQR: 1.25 – 5.00). The median near VA among patients aged 40 years and above was 4.0 Metric units (IQR: 1.60 – 6.30); while in those aged below 40 years it was 2.0 Metric units (IQR: 1.00 – 3.20).

Colour vision was normal in 143 (74.1%) patients, while 37 (19.2%) patients had nystagmus.

The commonest cause of low vision was retinitis pigmentosa in 32 (16.6%) patients. The causes of low vision are presented in Table 2. In majority (87%) of the patients the cause of low vision involved posterior segment disease.

**Table 2:** Causes of low vision

**Cause of low vision**	**Frequency (n)**	**Percent (%)**
Retinitis pigmentosa	32	16.6
ARMD^¶^	28	14.5
Optic atrophy	23	11.9
Glaucoma	22	11.4
Albinism	19	9.8
Non-specific maculopathy	16	8.3
Bilateral macular scars	14	7.3
Amblyopia	9	4.7
Undetermined	8	4.1
Corneal opacity	7	3.6
Degenerative myopia	7	3.6
Macular hole	3	1.6
Others*	5	2.6
Total	193	100.0

ARMD - Age related macular degeneration.

*Others: Diabetic retinopathy – 2, Rubella retinopathy – 1, Chronic uveitis – 1, Coloboma −1.

The commonest causes of low vision among children were albinism and optic atrophy, each accounting for 24.4%. Among the adults aged 16 – 64 years, the commonest cause was retinitis pigmentosa, occurring in 26.4% of patients. While in the elderly (65+ years), ARMD (45.2%) was the commonest.

**Figure 1:**
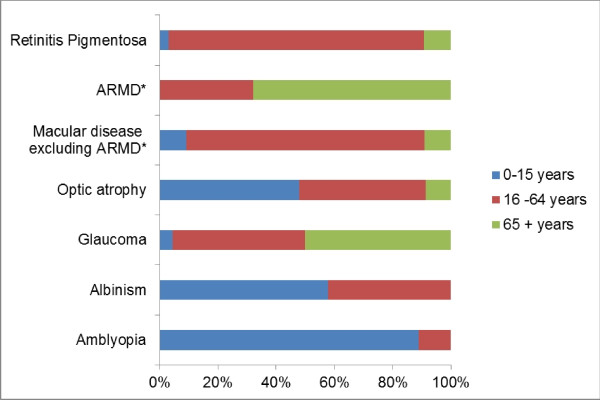
Frequency of different age groups within the major causes of low vision. *ARMD - Age related macular degeneration.

Most (87.5%) of the patients with retinitis pigmentosa were aged between 16 and 64 years; 11 (57.9%) of those with albinism were children aged less than 16 years, while majority (67.9%) of patients with ARMD were elderly (65+ years). The relative frequency of the three different age groups within each of the major causes of low vision is presented in Figure 1.

## Discussion

This study has presented data from a population of low vision clinic patients. A major advantage of low vision clinic studies when compared with population surveys, blind school studies or blind register studies is that they provide more reliable and usually detailed ophthalmic information about people with low vision [11,12]. However, such information may be rather clinic-specific and be strongly influenced by the sources of referral as well as the acceptance and utilisation of low vision services by the population served by the particular low vision clinic. In addition, the sample size of such studies is often limited as was the case in our study. Thus, they are prone to sampling errors and are limited in their extrapolation to the general population [13]. Notwithstanding, the information obtained from such studies can be very useful for planning low vision services, active care and rehabilitation [6].

The age distribution of our patients is different from previous reports from developed countries but is similar to those from other developing countries. Although the incidence of low vision has been reported to increase with age [12], in our study, a significant proportion (38.9%) of patients was below 30 years and majority (58%) were below 50 years of age, while less than a third were aged 60 years and above. This depiction of a younger population is similar to findings from Malaysia [14], Korea [6], Nepal [15], and India [16] in which the proportion of patients aged below 50 years were 74%, 69%, 58% and 68% respectively. In these developing countries, the proportion of low vision patients aged 60 years and above ranged between 16% and 26%.

On the other hand, in studies from developed countries, Leat and Rumney [17] (United Kingdom) found 77% of their patients to be aged 60 years and above; Elliot et al. [11] (Canada) reported that 66% of patients were 70 years or older; while in Australia, Wolffsohn and Cochrane [12] observed that 87% of patients were aged 60 years and above. This difference in the pattern of the age distribution may be a reflection of the older general populations in developed countries [11,14] and low life expectancy in developing countries [16]. Indeed, the proportion of the elderly (65 years and above) within the general population of Nigeria is only 3.3%, based on figures from the 2006 census [18]. Despite this fact, the difference may actually be an indication that the older population in developing countries are less likely to access and utilise low vision services than those in developed countries as a result of lower literacy and a relative lack of interest in reading.

The relatively high male to female ratio in our study is similar to that of other studies conducted in developing countries as follows: Korea- 1.8:1 [6], Malaysia- 2.2:1 [14], Nepal- 2.3:1 [15], and India- 2.6:1 [16]. It is, however, different from the pattern in developed countries where more females were found to present for low vision services [11,12,19]. This probably demonstrates the reduced access and utilisation of eye care services by females in developing countries [20,21].

In addition, it has been reported that the female predominance observed in studies from developed countries becomes more noticeable with age and may be related to greater longevity in women [12]. In our study, however, we found that the proportion of females reduced with age, though this trend was not statistically significant. Further research into the gender distribution among low vision clinic patients may shed more light on this observation.

Majority of our patients considered their problems with near and distance vision to be of equal importance. However, elderly patients were more likely to deem near vision as being their major problem; while children had a tendency to judge distance vision as more important. This observation perhaps portrays the additional effect of presbyopia on low vision in the elderly, although it may also signify that the elderly have a greater likelihood of central visual loss from macular disease.

Posterior segment disease accounted for the majority of causes of low vision in this study. This correlates with findings of most low vision clinic studies [6,11,12,14-17]. One difference, however, is that some previous reports found age related macular degeneration (ARMD) to be the commonest cause [11,12,17,19], while retinitis pigmentosa was the commonest in our study. Despite this difference, we found ARMD to be the second commonest cause, occurring in one out of every seven subjects seen in our low vision clinic. Similarly, in the Nigerian National blindness survey, ARMD was the third most common cause of low vision, accounting for 11.0% of subjects with low vision. Besides, there are other reports, specifically from developing countries, in which ARMD was also not the commonest cause [6,14-16]. Possible reasons for lower prevalence of ARMD in developing countries may include nutritional factors, less cigarette smoking, and lower body mass index (BMI) [22]. Further research is required for a better understanding of the role these factors in developing countries.

Retinitis pigmentosa has been found to be a major cause of low vision in only a couple of previous studies. Khan found it to be the commonest cause in a population of 410 low vision patients in India [16]. While Mohidin and Yusoff observed it to be the second commonest cause in a Malaysian low vision clinic [14]. Further research is necessary to investigate this finding in our patients in order to elucidate a possible explanation.

Glaucoma occupied the fourth position as a cause of low vision in our study population (11.4%) in contrast to findings of the Nigerian National Blindness and Visual Impairment Survey in which glaucoma was the most common (26.6%) cause of functional low vision [3]. The small sample size of our study may account for this difference. It may, however, be suggestive of poor uptake of low vision services by patients with glaucoma. Some glaucoma patients needing low vision care may not have been referred to the low vision clinic and may therefore have been missed. It is thus necessary to educate eye care providers and glaucoma patients about the available options of low vision assessment and low visual aids.

The low frequency of diabetic retinopathy as a cause of low vision in our study is contrary to findings from most of the previous reports from both developing and developed countries in which it usually featured as the second or third commonest cause [6,11,12,14,16]. Our finding is, however, in keeping with the low prevalence of diabetic retinopathy as a cause of blindness observed during the Nigerian National Blindness and Visual Impairment Survey [23].

Contrary to the previous report by Richard [24] in which cataract was the most common cause of low vision, cataract was uncommon in our study because most cases of cataract were satisfactorily managed in the main eye clinic by surgery. The few patients with cataracts in our study either had another disease as the primary cause of low vision (e.g. retinitis pigmentosa) or could not be operated because of poor health or other social reasons.

The major causes of low vision within the different age groups as shown in Figure 1 are quite similar to other reports [12,14]. As expected, congenital and heritable conditions were more common in children, while age related diseases were predominant in the elderly patients.

## Conclusions

It appears that the demographic and clinical characteristics of low vision patients in our setting are similar to that of patients in other developing countries, but different from those in developed countries. In addition, elderly patients and females may be under-utilising low vision services. We recommend that primary eye care practitioners including optometrists and general medical practitioners in Nigeria and other developing countries should be encouraged to be more mindful about identifying patients with low vision, especially females and the elderly and promptly referring such patients for low vision assessment. Our study would suggest that the current need for low vision care should be directed at providing succour for adults with retinitis pigmentosa, age related macular degeneration, optic atrophy, glaucoma and children with albinism.

More extensive multicentre research on the characteristics of low vision patients as well as the determinants of utilisation of low vision services is necessary to provide more data that would be useful for future planning and delivery of services.

## Competing interests

The authors declare that they have no competing interests.

## Authors’ contributions

CB conceived and designed the study. BO, GO and WI monitored data collection. BO performed analysis of the data and drafted the manuscript. CB, GO and WI contributed to the review of the manuscript. All authors read and approved the final manuscript.

## Pre-publication history

The pre-publication history for this paper can be accessed here:

http://www.biomedcentral.com/1471-2415/12/31/prepub
